# Increased Risk of Cognitive Decline in APOE ε2 Homozygotes with African Local Ancestry: Insights from a Brazilian Population

**DOI:** 10.1002/alz70855_107131

**Published:** 2025-12-25

**Authors:** Gabriel do Nascimento Santos, Marvin A.L.S. Alexandria, Lucas Aurélio Veronezz, Isadora Bianchi Ignácio, Samantha LG Paço, Claudia Kimie Suemoto, Renata Elaine Paraizo Leite, Michel Satya Naslavsky, Mayana Zatz, LAMP4

**Affiliations:** ^1^ University of São Paulo, São Paulo, São Paulo, Brazil; ^2^ University of São Paulo, Sao Paulo, São Paulo, Brazil; ^3^ Universidade de São Paulo, São Paulo, Sao Paulo, Brazil; ^4^ Division of Geriatrics, University of São Paulo Medical School, São Paulo, São Paulo, Brazil; ^5^ Physiopathology in Aging Laboratory (LIM‐22), Department of Internal Medicine, University of São Paulo Medical School, São Paulo, São Paulo, Brazil; ^6^ Biobank for Aging Studies of the University of São Paulo, São Paulo, São Paulo, Brazil; ^7^ University of São Paulo Medical School, São Paulo, São Paulo, Brazil; ^8^ Division of Geriatrics, Department of Internal Medicine, University of São Paulo Medical School, São Paulo, São Paulo, Brazil; ^9^ University of Sao Paulo Medical School, São Paulo, Brazil; ^10^ Brazilian Brain Bank of the Aging Brain Study Group; University of São Paulo, São Paulo, Brazil; ^11^ Biobank for aging studies of the University of São Paulo, São Paulo, Brazil; ^12^ University of Sao Paulo Medical School, Sao Paulo, Brazil; ^13^ Biobank for Aging Studies of the University of São Paulo Medical School, São Paulo, Sao Paulo, Brazil; ^14^ Physiopathology in Aging Laboratory (LIM‐22), University of Sao Paulo Medical School, São Paulo, São Paulo, Brazil; ^15^ Biobank for Aging Studies of the University of São Paulo, São Paulo, Sao Paulo, Brazil; ^16^ Department of Pathology, University of São Paulo Medical School, São Paulo, São Paulo, Brazil; ^17^ Hospital Israelita Albert Einstein, São Paulo, Brazil; ^18^ Human Genome and Stem Cell Center, São Paulo, São Paulo, Brazil; ^19^ University of São Paulo, São Paulo, Brazil

## Abstract

**Background:**

Apolipoprotein E plays a critical role in lipid metabolism and is associated with cardiovascular disease, cognitive decline, and Alzheimer's disease. While the *APOE* ε4 allele is a well‐established risk factor for these conditions, the *APOE* ε2 allele is considered protective, though its homozygosity increases cardiovascular risk.

**Method:**

This study investigates whether *APOE* ε2 homozygous carriers with African local ancestry are at differential risk for cognitive conditions compared to ε2 heterozygous carriers and other *APOE* genotypes. Given the unique genetic diversity of the Brazilian population, we specifically examined the role of African local ancestry in *APOE* ε2 homozygotes, as this group may exhibit distinct risk profiles due to population‐specific genetic and environmental factors. We genotyped 1,589 individuals from a population‐based cohort in São Paulo, Brazil, derived from a brain bank at the University of São Paulo. Surprisingly, we observed that the frequency of the rare homozygous ε2/ε2 genotype was significantly higher in the brain bank sample, suggesting that it may confer an increased risk for carriers.

**Result:**

To assess deviations in genotype frequencies, we performed Hardy‐Weinberg equilibrium tests between the genotypes 2/2, 2/3, 2/4, 3/3, 3/4, and 4/4. The ε2/ε2 genotype showed significant Hardy‐Weinberg disequilibrium (X² (1; 0.05) = 3.841 > 1.601), indicating potential selective pressures or founder effects.

Using microarray data from a subgroup of 720 individuals, we explored the role of local ancestry (African, European, or Native American) in modulating the risk associated with *APOE* ε2 homozygosity. Local ancestry inference (LAI) revealed a Hardy‐Weinberg disequilibrium among homozygote African ancestry ε2/ε2 carriers, with 6 out of 9 individuals, showing significant deviation (X² (3; 0.05) = 7.815 > 7.441). These findings suggest that *APOE* ε2 homozygosity, particularly in carriers of African local ancestry, may confer differential risks for cognitive conditions.

**Conclusion:**

Our results highlight the importance of considering local ancestry in genetic studies of complex diseases, especially in admixed populations like Brazil. This study provides new insights into the role of *APOE* ε2 homozygosity and local ancestry in modulating disease risk, with potential implications for personalized medicine and public health strategies.

